# Supplementary material for: Synthesis and assembly of extended quintulene
Source: Nat Commun. 2020 Aug 7;11:3976. doi: 10.1038/s41467-020-17691-7 (PMC7414228; doi:10.1038/s41467-020-17691-7)
Supplement: Supplementary file 1 — Supplementary Information [file 41467_2020_17691_MOESM1_ESM.pdf]

Supplementary Information for "Synthesis and Assembly of Extended Quintulene"

Hao Hou[1+], Xin-Jing Zhao[1+], Chun Tang[1+], Yang-Yang Ju[1], Ze-Ying Deng[1], Xin-Rong Wang[1], Liu-Bin Feng[1], Dong-Hai Lin[1], Xu Hou[1], Akimitsu Narita[2], Klaus Müllen[2,3], Yuan-Zhi Tan[1]*

[1] State Key Laboratory for Physical Chemistry of Solid Surfaces, Collaborative Innovation Center of Chemistry for Energy Materials, and Department of Chemistry, College of Chemistry and Chemical Engineering, Xiamen University, Xiamen 361005

[2] Max Planck Institute for Polymer Research, Ackermannweg 10, 55128, Mainz, Germany

[3] Institute of Physical Chemistry, Johannes Gutenberg-Universitat Mainz, Duesbergweg 10-14, 55128 Mainz, Germany

+These authors contributed equally to this work

*Corresponding author. Email: yuanzhi_tan@xmu.edu.cn (T.Y.-Z.)

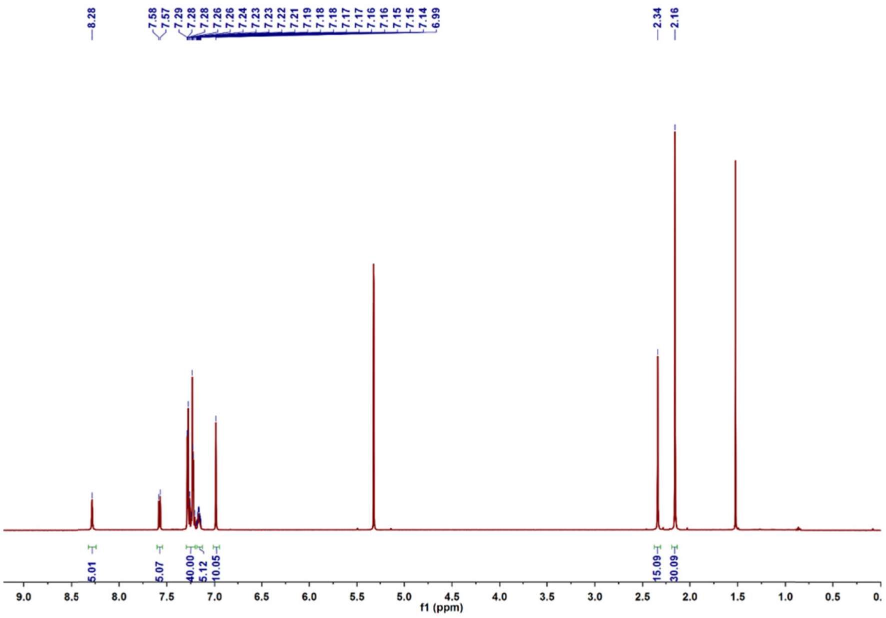

Supplementary Figure 1. ^{1}H NMR spectrum of 2. (600 MHz, CD_2Cl_2)： δ = 8.28 (s, 5H), 7.58 (d, 6H), 7.29-7.21 (m, 40H), 7.19-7.14 (m, 5H) , 7.99 (s, 10H), 2.34 (s, 15H), 2.16 (s, 30H) ppm.

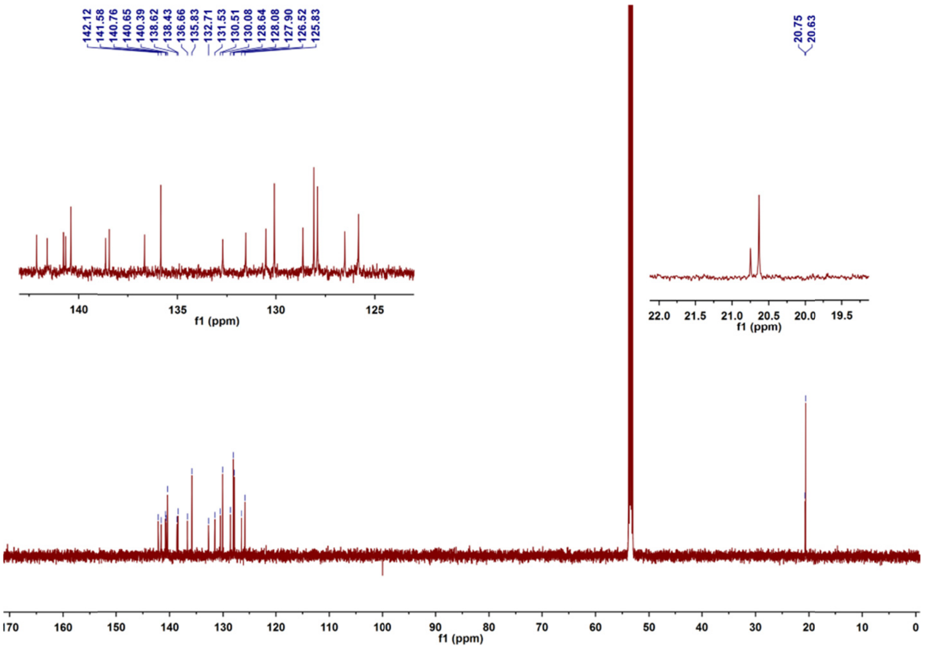

Supplementary Figure 2. ^{13}C NMR spectrum of 2. (150 MHz, CD_2Cl_2): δ = 142.12, 141.58, 140.76, 140.65, 140.39, 138.62, 138.43, 136.66, 135.83, 132.71, 131.53, 130.51, 130.08, 128.64, 128.08, 127.90, 126.52, 125.83, 20.75, 20.63 ppm

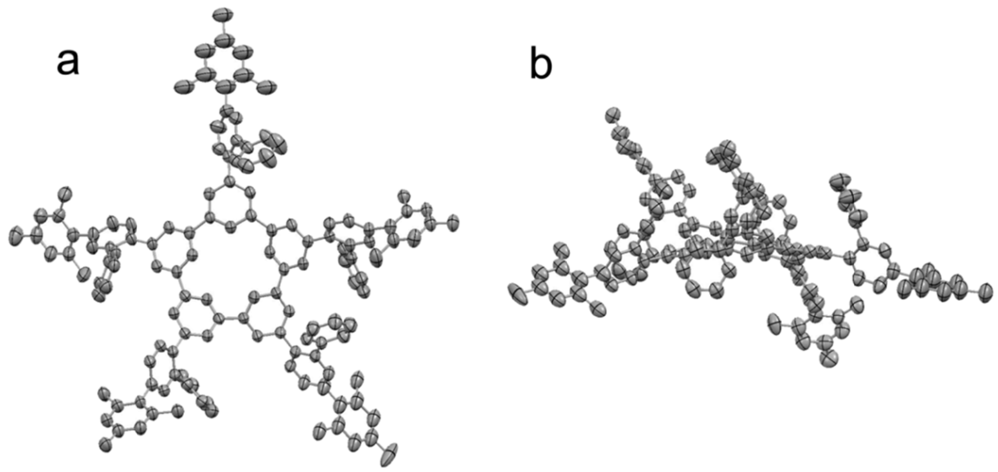

Supplementary Figure 3. Crystal structure of 2. a. top view, b. side view. The hydrogens were omitted for clarity.

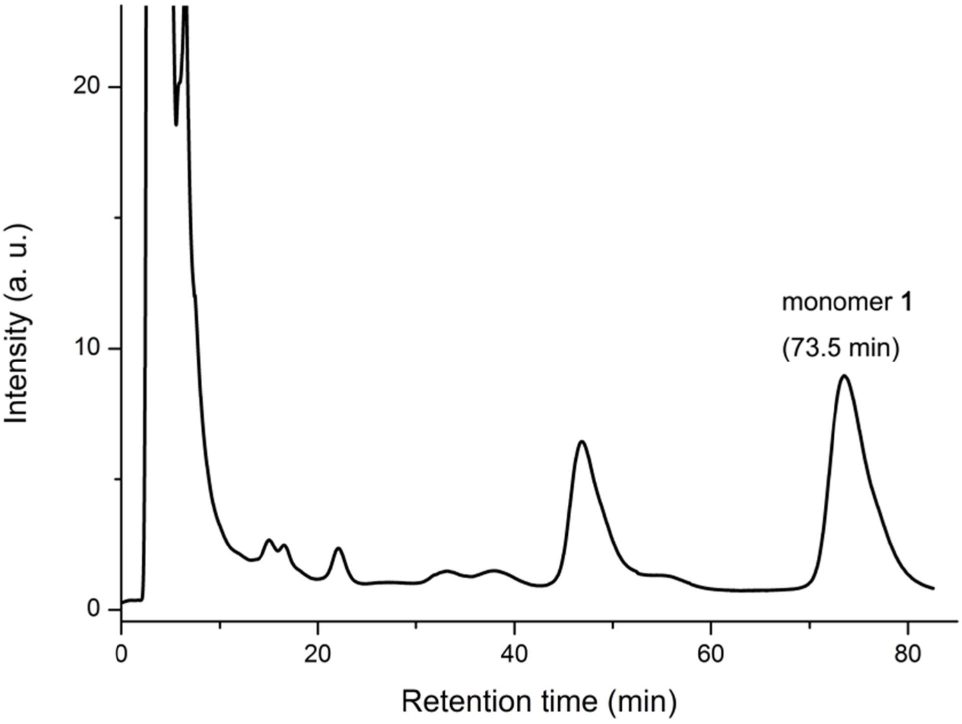

Supplementary Figure 4. HPLC chromatogram for the crude product of cyclodehydrogenation using a 5PBB HPLC column (I.D. 10 × 250 mm) at a flow rate of 4 mL/min with toluene as mobile phase.

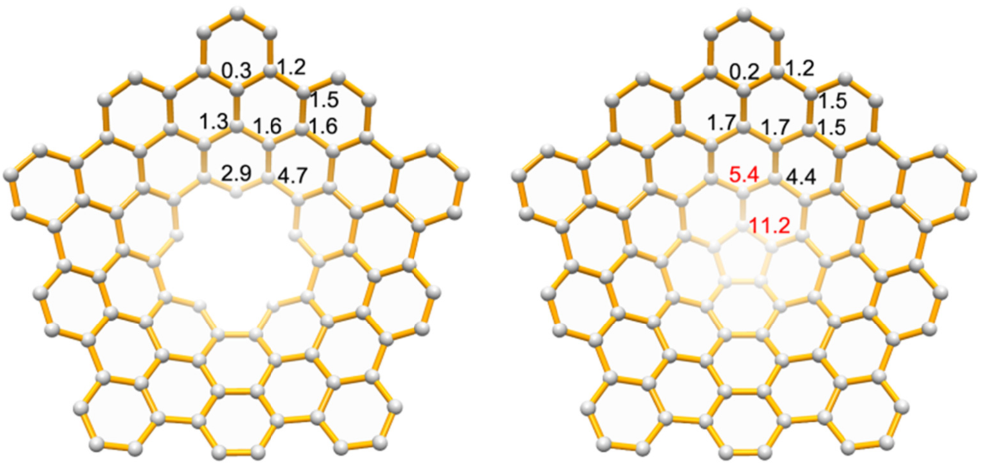

Supplementary Figure 5. The pyramidalization angle of the carbon atoms in 1 (left) and its counterpart without the cavity (right). The structures were optimized by DFT calculations.

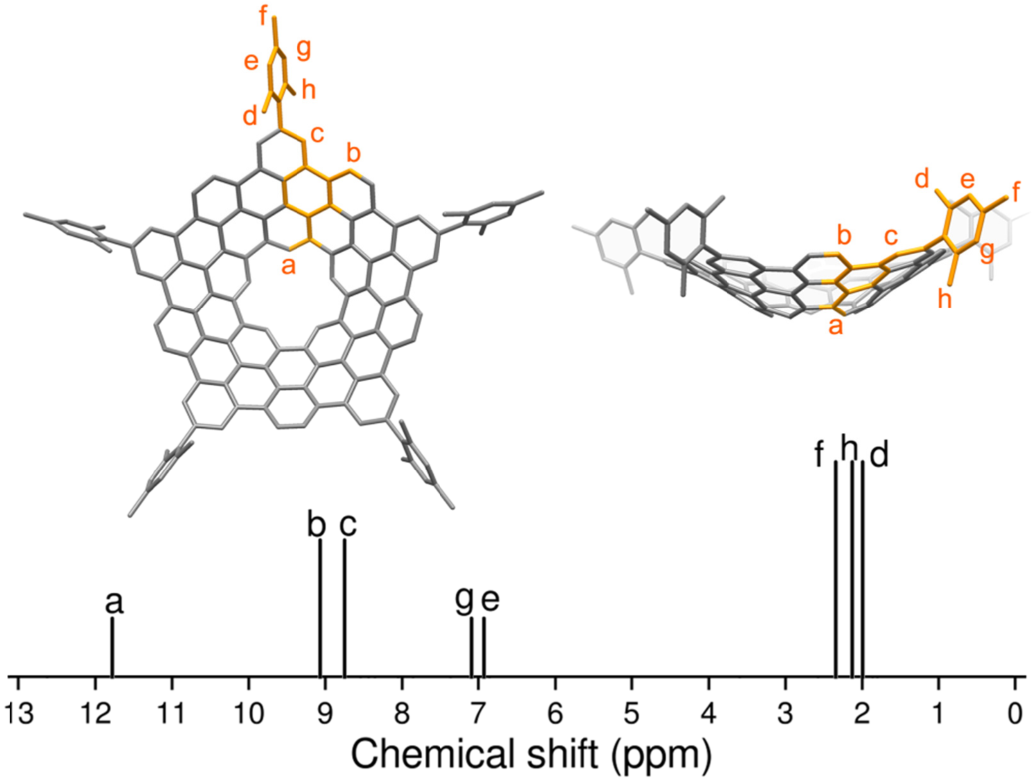

Supplementary Figure 6. Theoretically calculated ^{1}H NMR spectrum of 1. The number of protons corresponding to one peak was represented by the height of the lines.

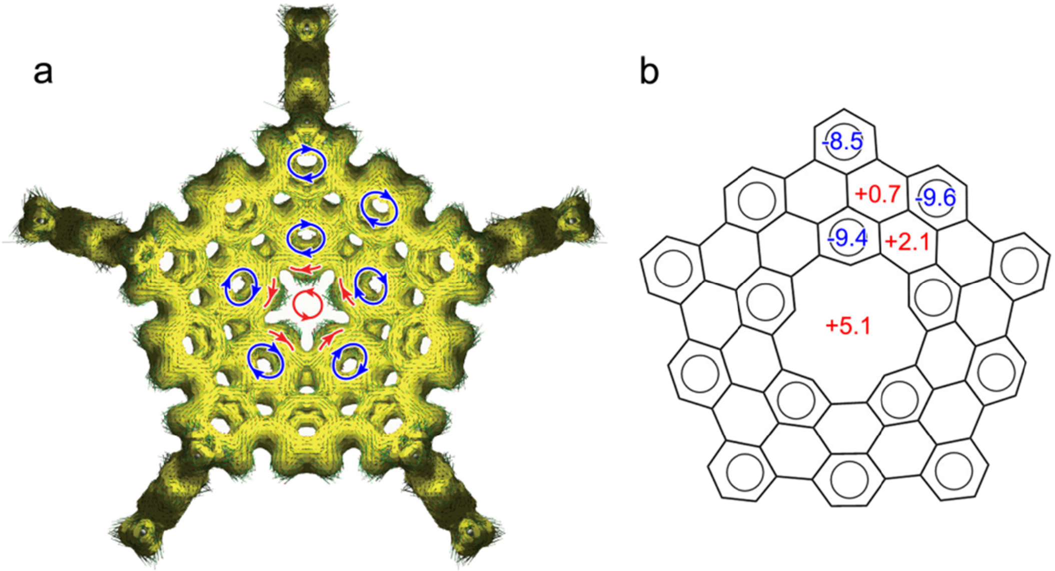

Supplementary Figure 7. Anisotropy of the induced current density (AICD) plot and NICS(0)$_{iso}$ of 1. a. AICD plot of **1**. Blue and red cycles represent diatropic and paratropic currents, respectively. The magnetic field is perpendicular to the mean plane of the molecule and points downwards. b. The NICS(0)$_{iso}$ values of **1** are shown at the corresponding positions. All the π electrons of **1** can be partitioned into Clar sextets. The positive and negative values are labelled in red and blue, respectively.

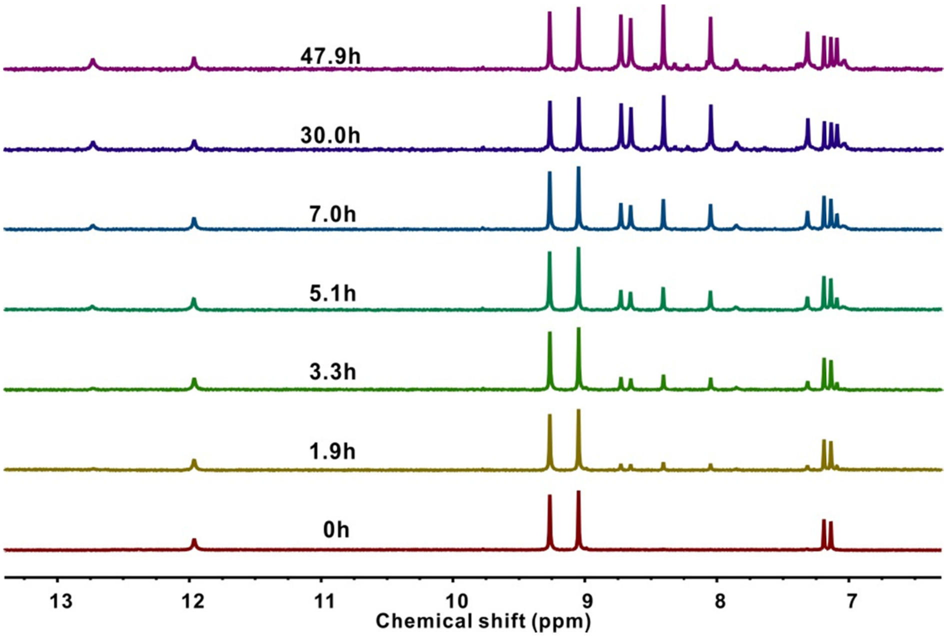

Supplementary Figure 8. Time-dependence of ^{1}H NMR spectra of 1 at 40 ^{o}C in $C_2Cl_4D_2$.

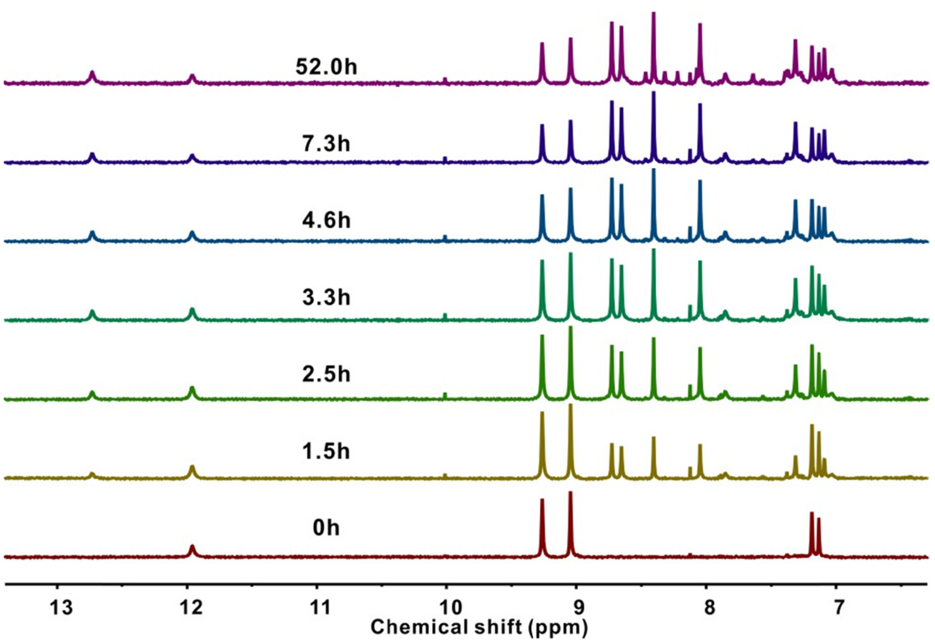

Supplementary Figure 9. Time-dependence of ^{1}H NMR spectra of 1 at 50 ^{o}C in $C_2Cl_4D_2$.

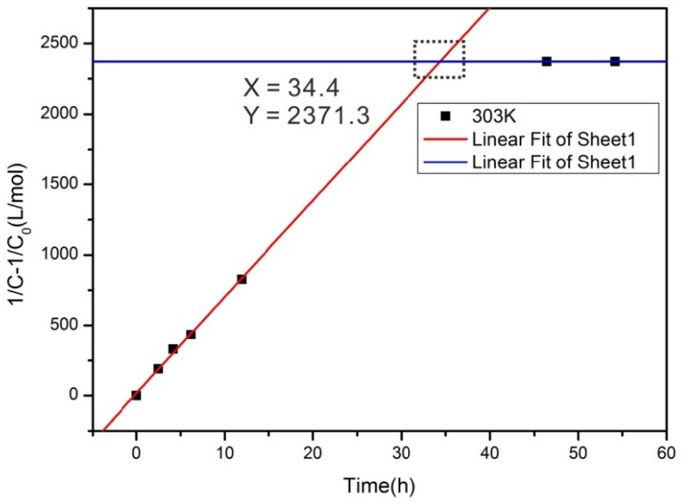

Supplementary Figure 10. The plot of ($1/C-1/C_0$) vs time at 30 ^{o}C measured in $C_2Cl_4D_2$. (C_0, the initial concentration of monomer; C, time-dependent concentration of monomer). Before equilibration, the time-dependent concentration of monomer (C) decreases with increasing reaction time. According to second-order kinetics, $1/C-1/C_0$ should increase linearly with time (red line) before equilibration. The experimental data fit the red line well, which validates the second-order kinetics for the assembly of extended quintulene. After equilibration, C remains unchanged, thus $1/C-1/C_0$ vs time should be a line parallel to the time axis (blue line). Then the intersection of the red

and blue lines is the point when chemical equilibrium is attained (34.4 h).

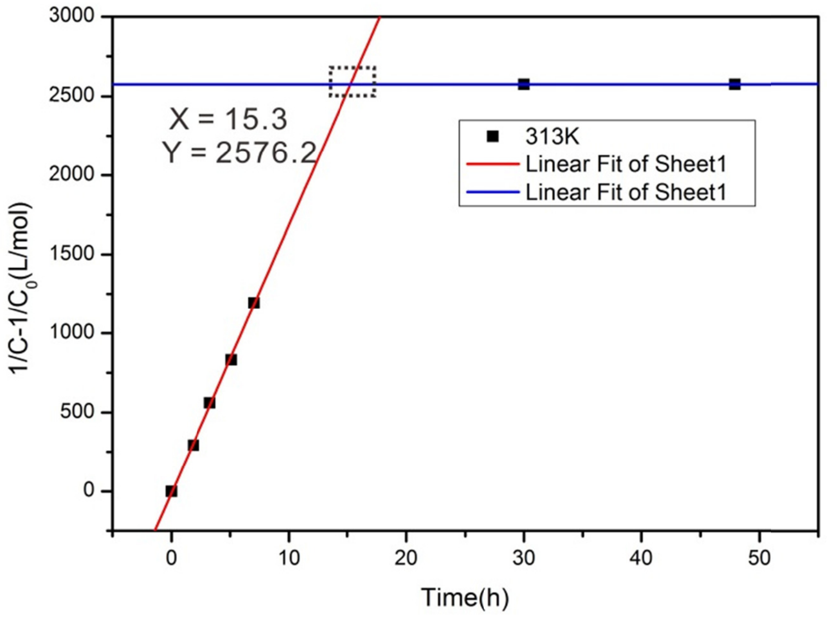

Supplementary Figure 11. The plot of (1/C-1/C_0) vs time at 40 °C measured in $C_2Cl_4D_2$. (C_0, the initial concentration of monomer; C, time-dependent concentration of monomer). According to the intersection of the red and blue lines, chemical equilibrium is attained after 15.3 h at 40 °C.

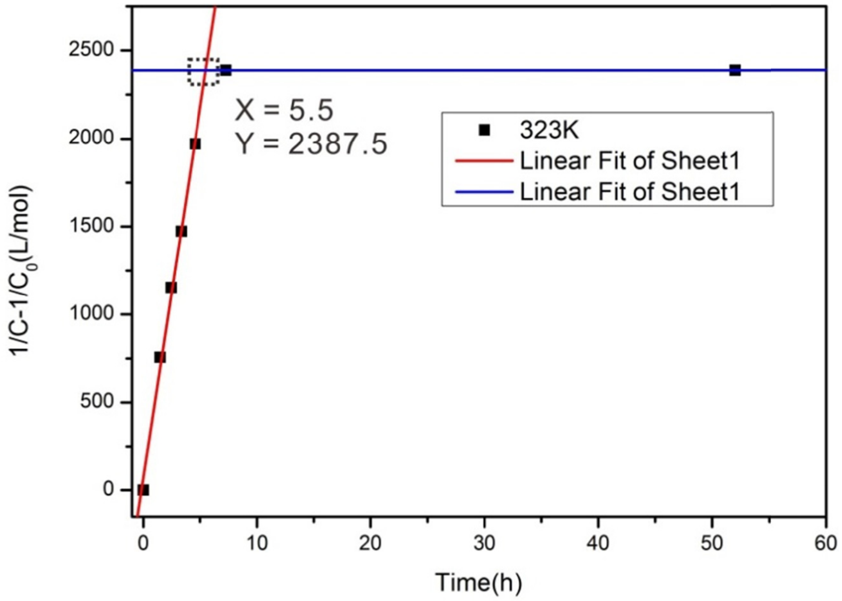

Supplementary Figure 12. The plot of (1/C-1/C_0) vs time at 50 °C measured in $C_2Cl_4D_2$. (C_0, the initial concentration of monomer; C, time-dependent concentration of monomer). According to the intersection of the red and blue lines, chemical equilibrium is attained after 5.5 h at 50 °C.

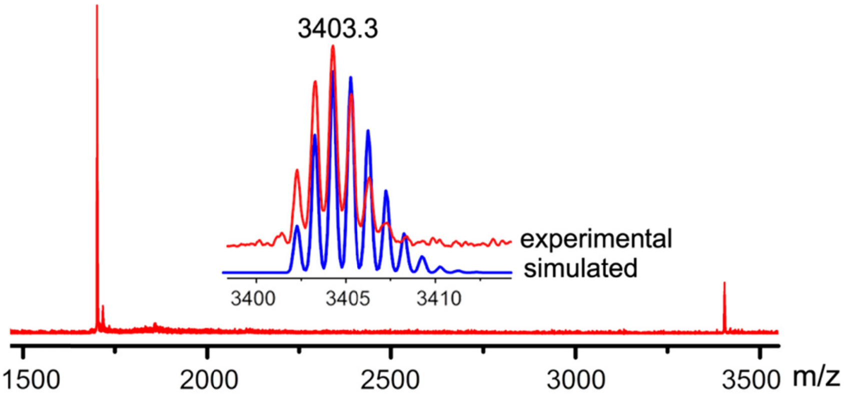

Supplementary Figure 13. Mass spectrum of equilibrated sample. The isotopic distribution for the mass peak of (**1**)$_2$ is shown as the insert figure.

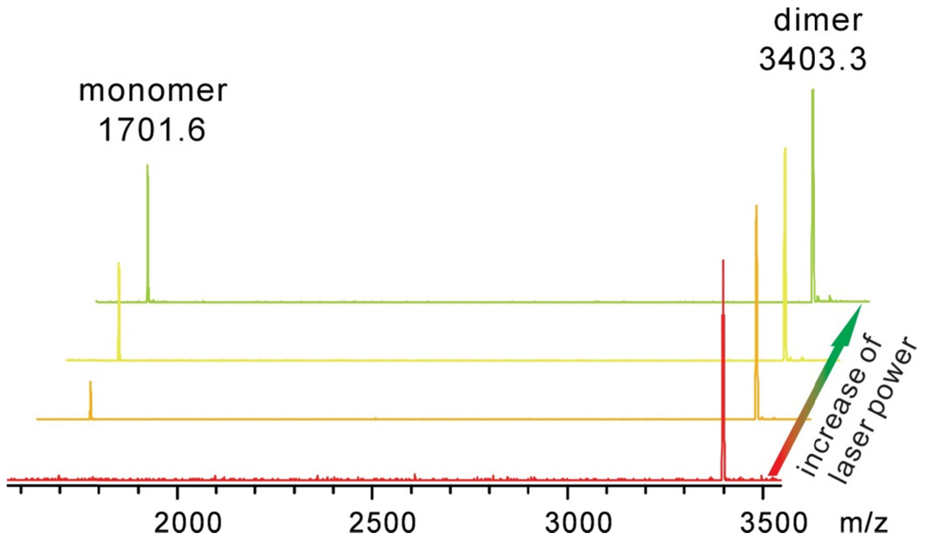

Supplementary Figure 14. Mass spectra of (1)$_2$ with different laser power.

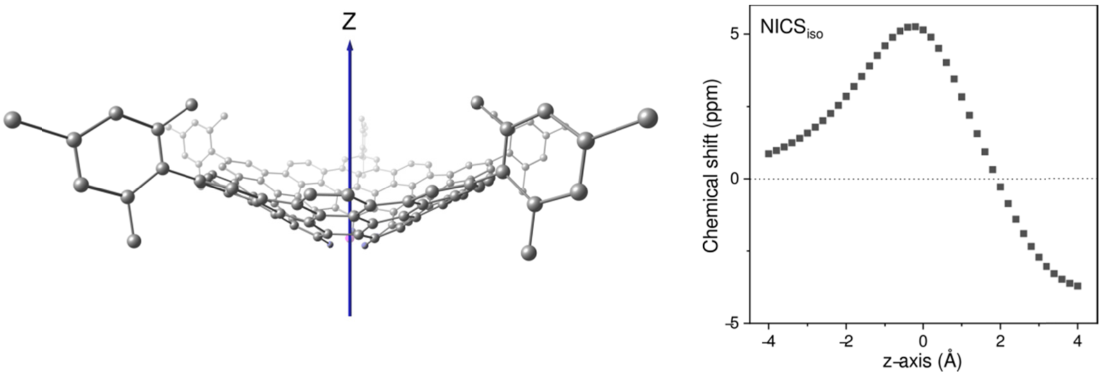

Supplementary Figure 15. NICS values along the quintuple axis (z-axis) of 1. The origin is set at the bottom center of **1** (pink dot).

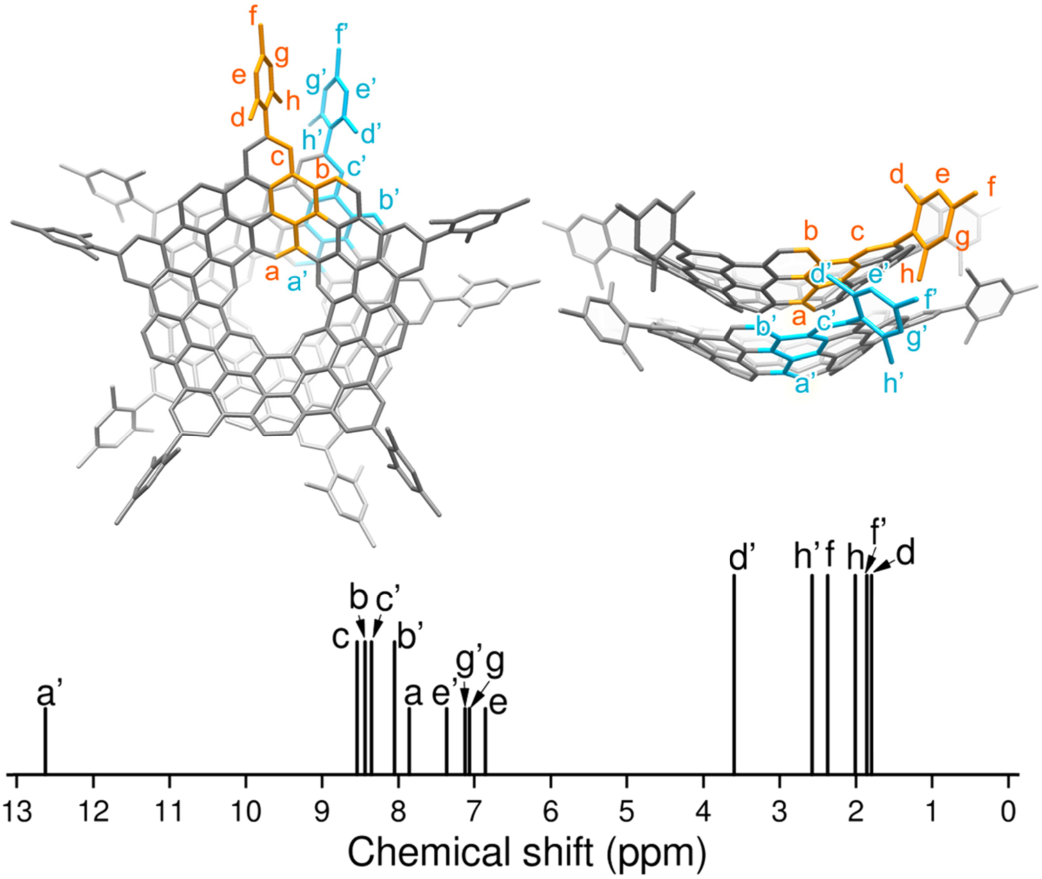

Supplementary Figure 16. Theoretically calculated ^{1}H NMR spectrum of $(1)_2$. The number of protons corresponding to one peak was represented by the height of the lines.

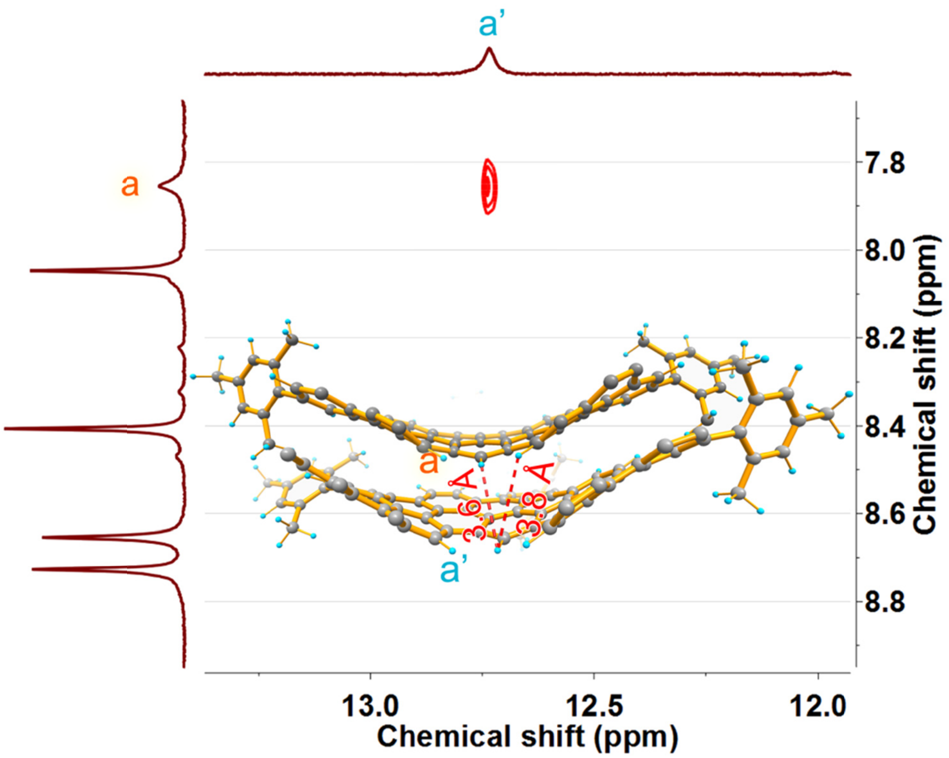

Supplementary Figure 17. Expanded 2D NOESY of (1)$_2$ showing the coupling between protons of inner cavity (H_a and $H_{a'}$). The H···H distance between hydrogens of the inner cavity found in (**1**)$_2$ is shown in the insert figure (3.6 and 3.8 Å), represented as red dashed lines

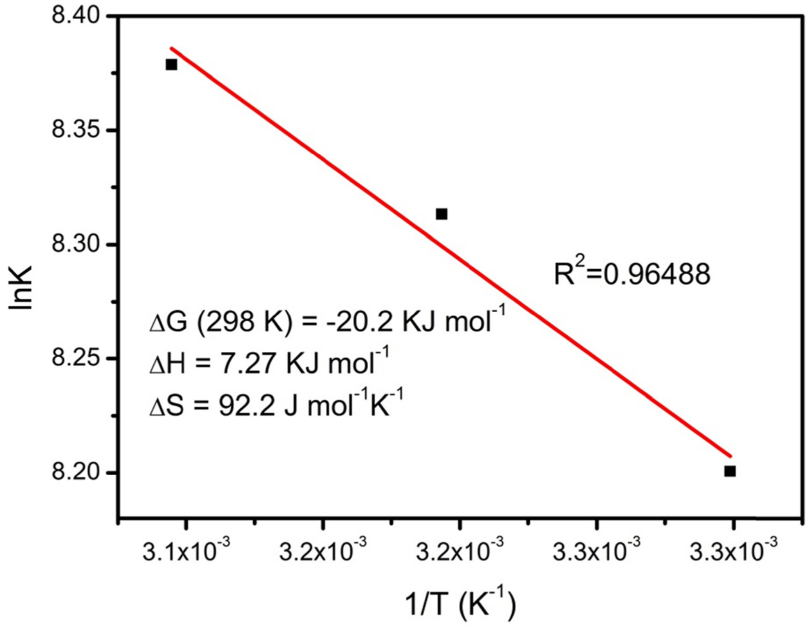

Supplementary Figure 18. Analysis of the dimerization in $C_2Cl_4D_2$ by a van't Hoff plot.

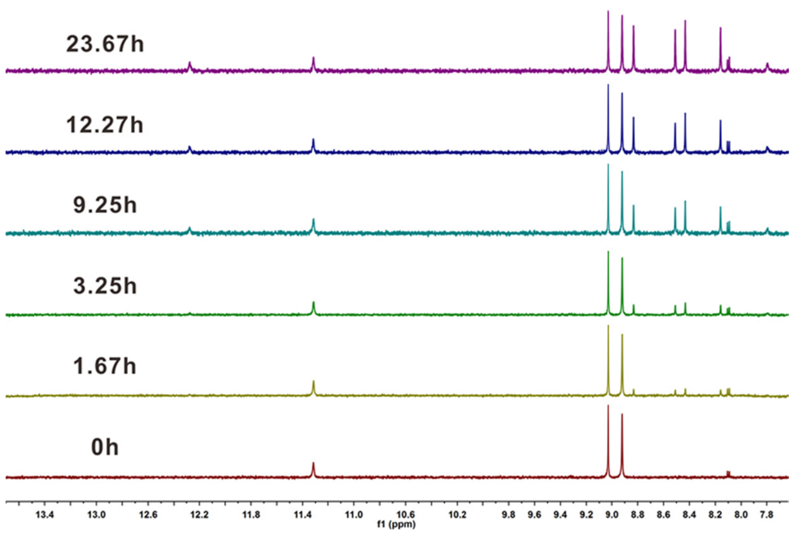

Supplementary Figure 19. Time-dependence of ^{1}H NMR spectra of 1 at 30 ^{o}C in C_6D_6.

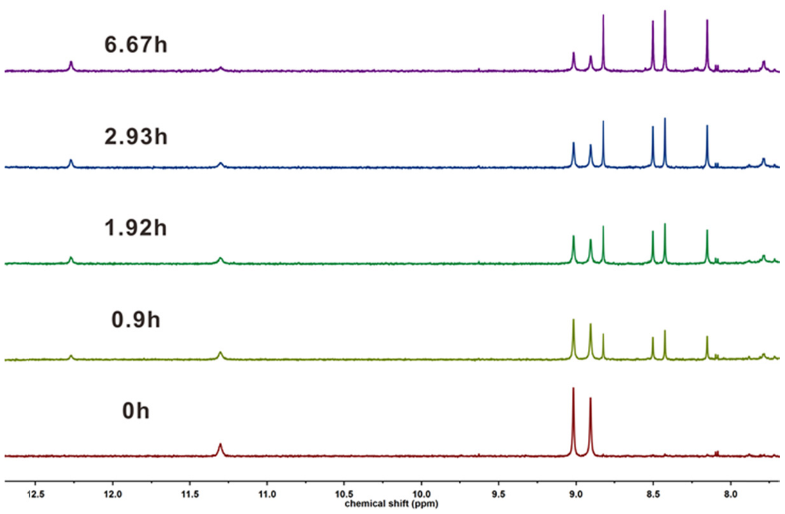

Supplementary Figure 20. Time-dependence of ^{1}H NMR spectra of 1 at 40 ^{o}C in C_6D_6.

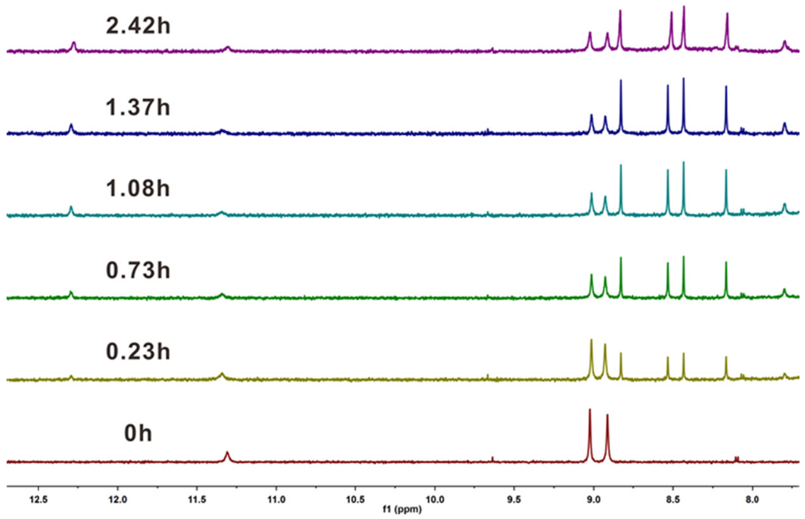

Supplementary Figure 21. Time-dependence of ^{1}H NMR spectra of 1 at 50 ^{o}C in C_6D_6.

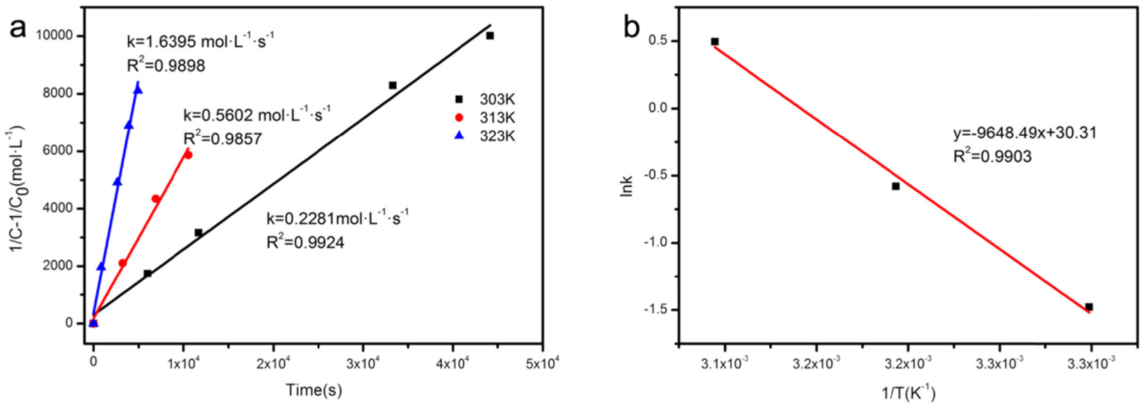

Supplementary Figure 22. Kinetics of dimerization measured in C_6D_6. a. Linear regression plot showing the second-order kinetics of the dimerization of **1**. b. Arrhenius plot for the dimerization of **1**, revealing an activation energy of 80.2±5.6 kJ mol^{-1}. (C_0, the initial concentration of monomer; C, temporal concentration of monomer; *k*, reaction rate constant)

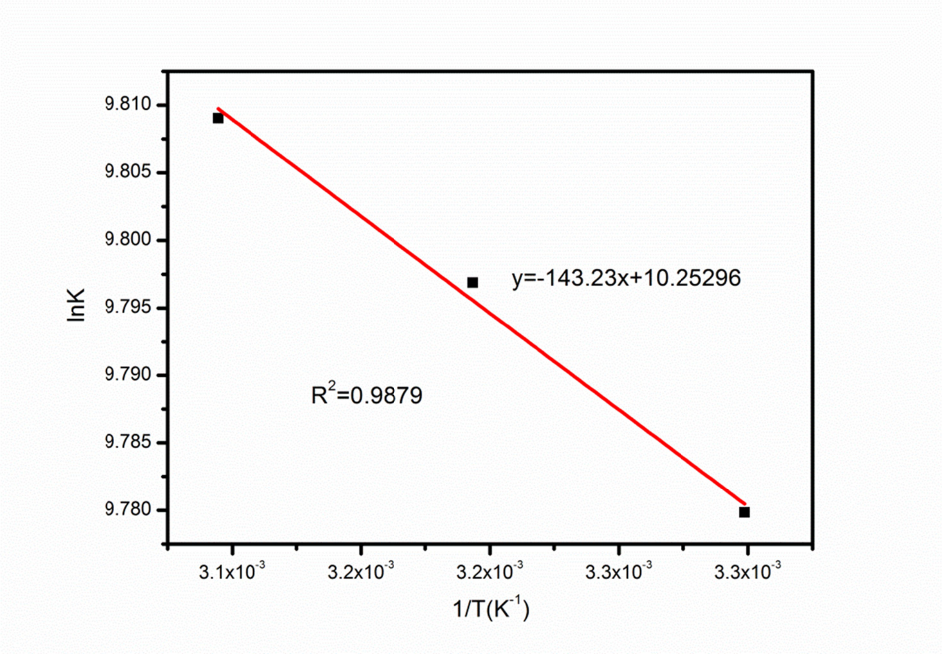

Supplementary Figure 23. Analysis of the dimerization in C_6D_6 by a van't Hoff plot, revealing a ΔH of 1.2±0.1 kJ·mol^{-1} and ΔS of 85.2±0.3 J·mol^{-1}K^{-1}.

Compound			
**1**	420 nm<br>(23809 cm^{-1})	445 nm<br>(22472 cm^{-1})	479 nm<br>(20877 cm^{-1})
(**1**)$_2$	417 nm<br>(23980 cm^{-1})	451 nm<br>(22172 cm^{-1})	485 nm<br>(20619 cm^{-1})

Supplementary Table 1. UV-Vis absorption parameters of **1** and (**1**)$_2$.

Compound					
**1**	500 nm	520 nm	536 nm	560 nm	575 nm
(**1**)$_2$	509 nm	528 nm	543 nm	563 nm	584 nm

Supplementary Table 2. PL parameters of **1** and (**1**)$_2$.

Supplementary Note 1

NMR assignment for 1

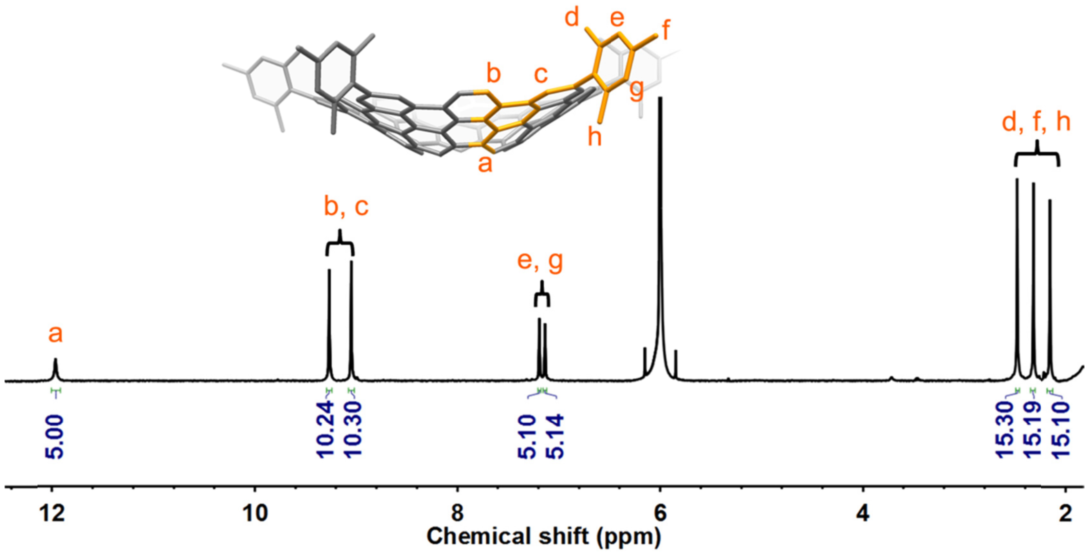

Supplementary Figure 24. ^{1}H NMR for 1. According to the chemical shifts, the signals are grouped into four categories. The asymmetric unit of **1** is highlighted in orange. (600 MHz, $C_2D_2Cl_4$): δ = 11.96 (s, 5H), 9.26 (s, 10H), 9.05 (s, 10H), 7.19 (s, 5H) , 7.14 (s, 5H), 2.48 (s, 15H), 2.32 (s, 15H), 2.15 (s, 15H) ppm.

First, the peak at 11.96 ppm can be easily assigned to the proton of the inner cavity, according to its chemical shift and integration. The peaks at 9.26 and 9.05 ppm are assigned to the protons at the periphery of the aromatic core, due to their larger chemical shift compared with the hydrogens at the benzene rings. The peaks at 7.19 and 7.14 ppm are due to the protons at the benzene ring of mesityl groups. The peaks at 2.48, 2.32 and 2.15 ppm correspond to the methyl groups of the mesityl substituents. The intensity ratio of all peaks matches the C_{5v} molecular symmetry of **1** well (Supplementary Figure 24).

The further signal assignments for **1** are achieved with the assistance of NOESY. Based on the NOESY correlation between protons at the periphery of the aromatic core and those of the methyl groups (Supplementary Figure 25), the peak at 9.05 ppm is assigned to H_c, due to its stronger coupling with the methyl groups. Consequently, the peak at 9.26 is H_b.

The para-methyl (H_f) protons of the mesityl groups are far away from the protons at the periphery of inner core (H_c and H_b) and do not show any NOE coupling with H_c and H_b. Thus, we can assign the peak at 2.48 ppm to H_f. This assignment of H_f is in consistence with the analysis based on the proton coupling between protons (H_e and H_g) at the benzene ring and those (H_d, H_h and H_f) of methyl protons of the mesityl groups (Supplementary Figure 26).

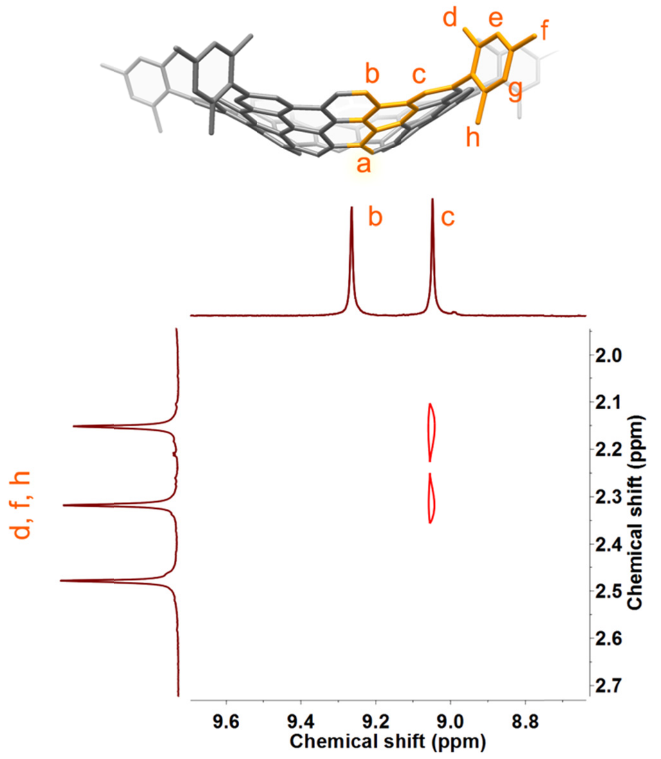

Supplementary Figure 25. Expanded 2D NOESY of 1 showing the proton coupling between protons (H_b and H_c) at the periphery of aromatic core and protons of the methyl groups at mesityl substituents (H_d, H_f and H_h).

The NICS calculations indicate that protons inside the cone are shielded and those outside the cone are deshielded (Supplementary Figure 15), therefore, we assign the remaining alkyl signals (2.32 and 2.15 ppm) to H_h and H_d, respectively, which is consistent with the calculated NMR spectrum of **1** (Supplementary Figure 6). Then, we can assign the peaks at 7.19 and 7.14 ppm to H_e and H_g according to their NOE coupling with H_d and H_h (Supplementary Figure 26). The experimental assignment of all the protons in **1** is in good agreement with the theoretical NMR spectrum computed based on the structure of **1** (Supplementary Figure 6).

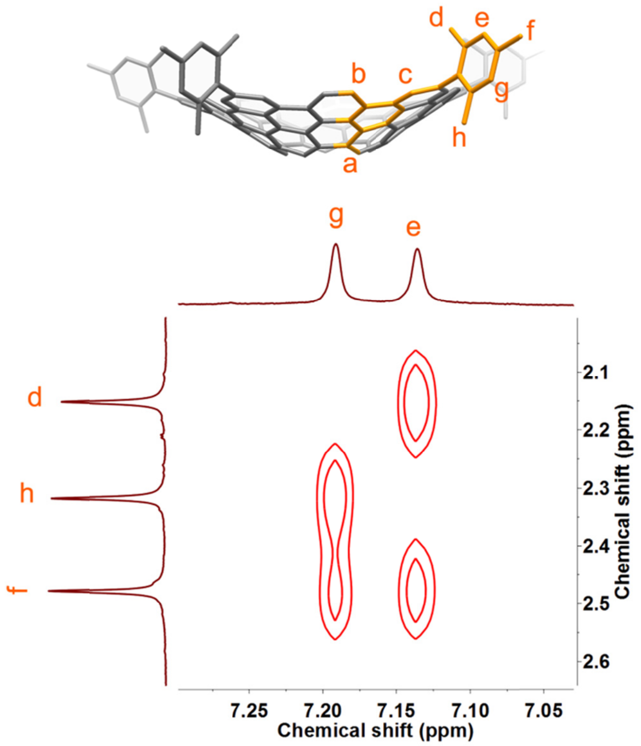

Supplementary Figure 26. Expanded 2D NOESY of 1 showing the proton coupling between protons (H_e and H_g) at benzene ring and those (H_d, H_h and H_f) at methyl groups. Only the protons at the para-methyl (H_f) groups can couple with both meta-protons at the benzene ring (H_e and H_g). Then the peak at 2.48 should be the signal of H_f.

Supplementary Note 2

NMR assignment for (1)$_2$

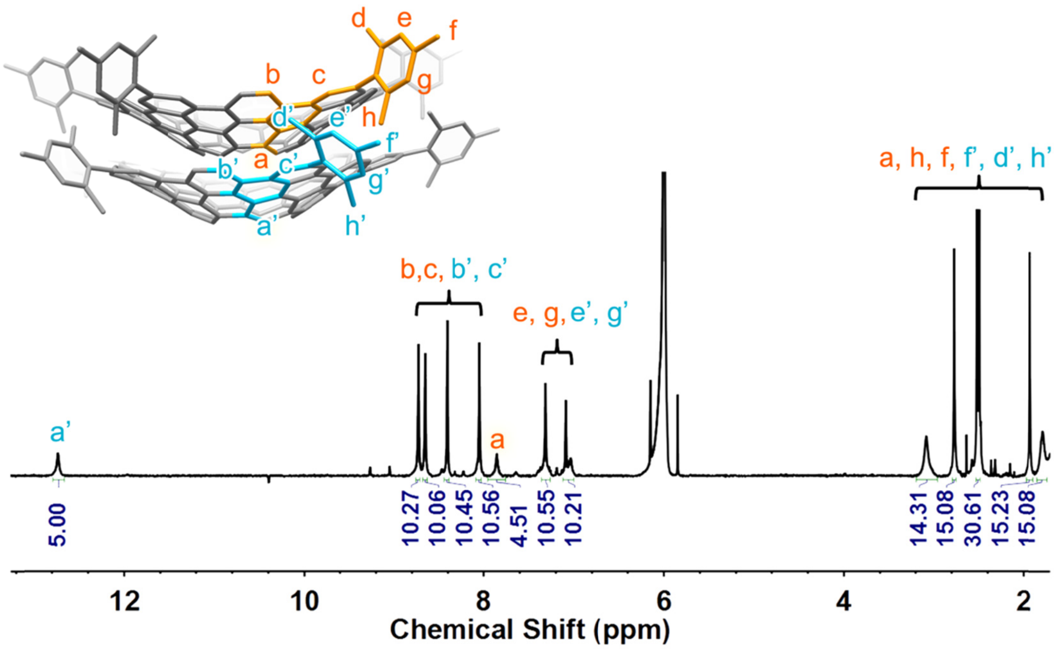

Supplementary Figure 27. ^{1}H NMR for (1)$_2$. According to the chemical shifts, the signals are grouped into four categories. (600 MHz, $C_2D_2Cl_4$): δ = 12.74 (s, 5H), 8.73 (s, 10H), 8.65 (s, 10H), 8.41 (s, 10H), 8.05 (s, 10H), 7.86 (s, 5H), 7.35-7.30 (s+s, 10H), 7.12-7.00 (s+s, 10H), 3.08 (s, 15H), 2.77 (s, 15H), 2.52 (s, 15H), 2.49 (s, 15H), 1.93 (s, 15H), 1.79 (s, 15H) ppm.

First, the NMR peaks of (**1**)$_2$ can be classified into four categories, i.e. protons at the inner cavity (12.74 ppm), at the periphery of the aromatic core (8.73, 8.65, 8.41, 8.05 ppm), at the benzene ring of mesityl groups (7.35-7.00 ppm) and at their methyl groups (3.08-1.79 ppm) (Supplementary Figure 27). Another signal of protons at the inner cavity is found at 7.86 ppm and assigned by NOESY correlation between protons at inner cavity from different layers (Supplementary Figure 17). Then according to NICS calculations (Supplementary Figure 15), the peak at 12.74 ppm is assigned to $H_{a'}$ and the peak at 7.86 is assigned to H_a. As shown in the theoretically calculated NMR spectrum of (**1**)$_2$ (Supplementary Figure 16), the signals of inner protons are at 12.63 ppm for $H_{a'}$ and 7.85 ppm for H_a, in good agreement with

experimental results.

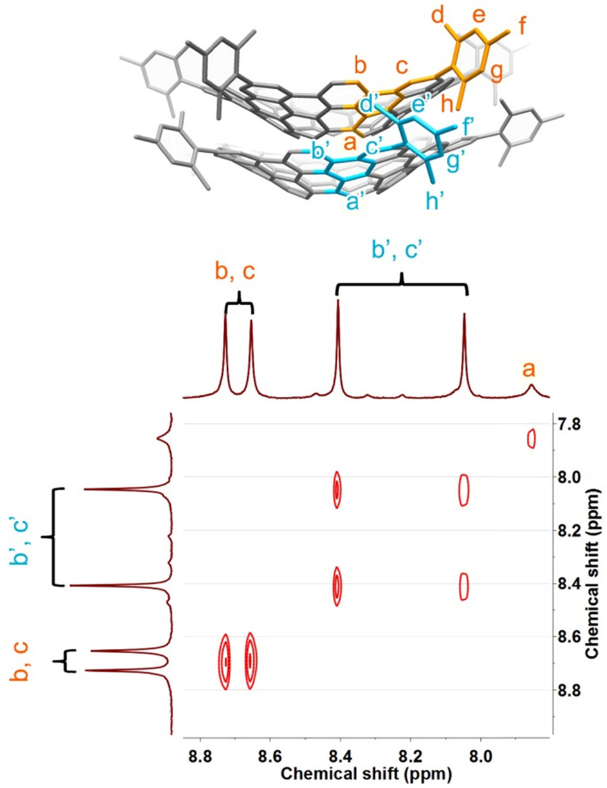

Supplementary Figure 28. Expanded 2D NOESY of (1)$_2$ showing the coupling between peripheral protons of the aromatic core. The insert is the asymmetric unit of (**1**)$_2$.

According to NOE signals between the protons at the periphery of aromatic core in (**1**)$_2$ (Supplementary Figure 28), we can group signals at 8.73, 8.65 ppm to the peripheral protons of one layer of (**1**)$_2$, signals at 8.41, 8.05 ppm to another layer. The distribution of these signals fits the assignment of DFT-calculated NMR spectrum (Supplementary Figure 16). With the help of DFT-calculated NMR spectrum (Supplementary Figure 16), we can further assign peaks at 8.73 and 8.65 ppm to H_b and H_c, peaks at 8.41 and 8.05 ppm to $H_{b'}$ and $H_{c'}$.

The methyl groups at the para-position of mesityl substituents (H_f and $H_{f'}$) are far away from the aromatic core and cannot couple with the protons of the aromatic core, as shown in Supplementary Figure 29. Thus, peaks at 2.52 and 2.49 ppm were assigned to H_f and $H_{f'}$.

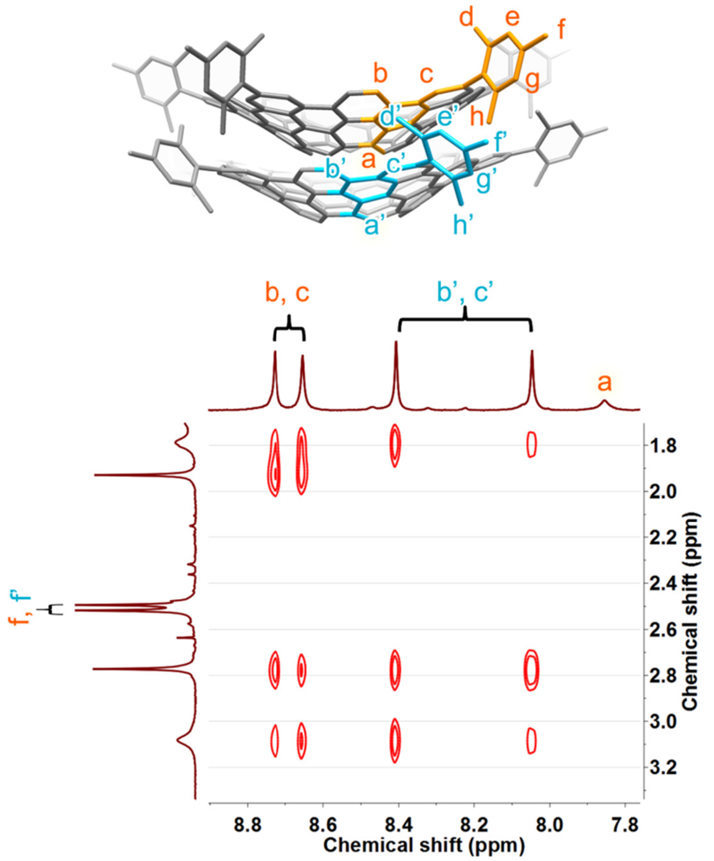

Supplementary Figure 29. Expanded 2D NOESY of (1)$_2$ showing the coupling between protons at the periphery of the aromatic core and methyl protons of mesityl groups. The insert is the asymmetric unit of (**1**)$_2$.

The methyl groups at the ortho-positions of mesityl groups are assigned by NOE coupling between methyl groups (Supplementary Figure 30). Due to the bilayer structure of (**1**)$_2$, H_h and $H_{d'}$ points toward the interlayer spacing of (**1**)$_2$ and are spatially close (Supplementary Figure 30). The other methyl groups from different layers in (**1**)$_2$ are far away. As shown in Supplementary Figure 30, only NOE coupling signals between the peaks at 3.08 and 1.79 ppm are found, which indicates that they are the signals of $H_{d'}$ and H_h. Then we assign the peak at 3.08 ppm to $H_{d'}$ and the peak at 1.79 ppm to H_h with the help of calculations (Supplementary Figure 30). In the same way, the remaining peaks at 2.77 and 1.93 ppm can be assigned to $H_{h'}$ and H_d, respectively. Peaks at 7.35-7.30 (s+s, 10H) and 7.12-7.00 (s+s,10H) are assigned to H_e, H_g and $H_{e'}$ and $H_{g'}$, respectively, with the help of the DFT-calculated NMR spectrum.

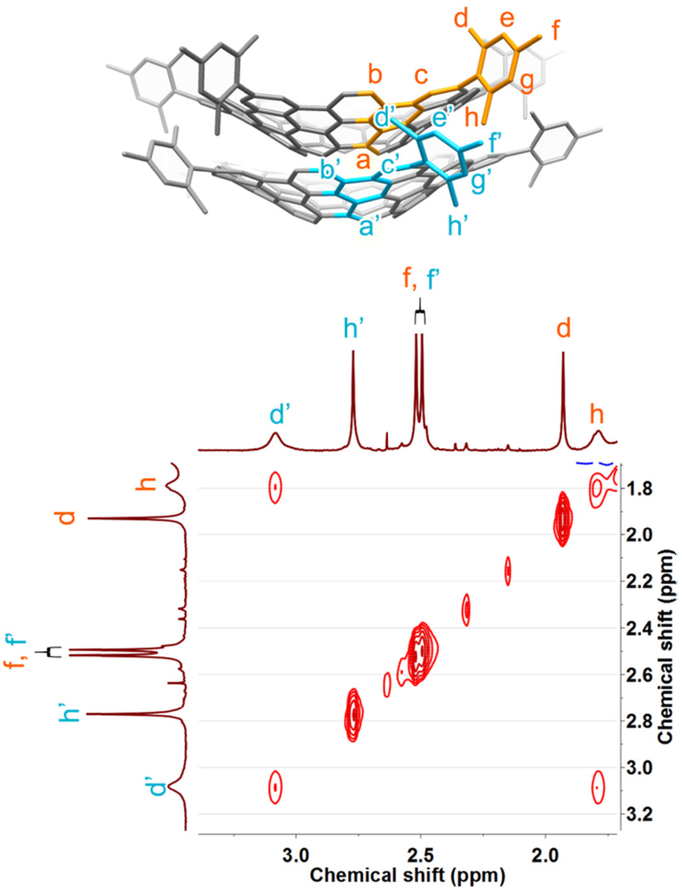

Supplementary Figure 30. Expanded 2D NOESY of (1)$_2$ showing the proton coupling between methyl protons of mesityl groups. The insert is the asymmetric unit of (**1**)$_2$.

Supplementary Note 3

Crystallographic information

The crystals of **2** were measured on a Rigaku Oxford SuperNova Diffractometer while the temperature of the crystal was controlled by Oxford Cryostream 700. Using Olex2[1], all the initial structures were solved with the SHELX-XT structure solution program using the direct method and refined with the XL refinement package using Least Squares minimization.

Crystallographic data were deposited in the Cambridge Crystallographic Data Centre (CCDC 1974252). The data can be obtained free of charge from the Cambridge Crystallographic Data Centre via www.ccdc.cam.ac.uk/data_request/cif.

Crystal Data for **2**: trigonal, trigonal, space group R-3 (no. 148), a = 60.318(2) Å, c = 18.9848(5) Å, V = 59818(5) Å^3, Z = 6, T = 99.98(19) K, $\mu(CuK_\alpha)$ = 0.367 mm^{-1}, D_{calc} = 0.866 g/cm^3, 50316 reflections measured, 21126 unique (R_{int} = 0.0633, R_{sigma} = 0.0819) which were used in all calculations. The final R_1 was 0.1192 (I > 2σ(I)) and wR_2 was 0.3440 (all data).

Supplementary Note 4

Density functional theory calculation

Density functional theory (DFT) calculations were carried out using the Gaussian 16 program package.[2] The models of **1** and (**1**)$_2$ were optimized at the hybrid meta exchange-correlation M06-2X functional[3] with 3-21G basic set[4]. The counterpart of **1** without the cavity was optimized at UM06-2X functional with 3-21G basic set.

The ^{1}H-NMR spectra of monomer **1** and dimer (**1**)$_2$ (red lines in Figure 3c and 5c, Supplementary Figures 6 and 16) were calculated for comparison with the experimental NMR to assist in resolving the structures of **1** and (**1**)$_2$. These calculations were performed using the GAUSSIAN 16 program package. The B3LYP functional[5-6] and the 6-31G* basis sets[7-8] were employed to calculate the NMR of **1** and (**1**)$_2$.

Supplementary references

1. Dolomanov, O. V.; Bourhis, L. J.; Gildea, R. J.; Howard, J. A. K.; Puschmann, H., OLEX2: a complete structure solution, refinement and analysis program. *J. Appl. Cryst.* **2009,** *42*, 339-341.
2. Frisch, M.; Trucks, G.; Schlegel, H.; Scuseria, G.; Robb, M.; Cheeseman, J.; Scalmani, G.; Barone, V.; Petersson, G.; Nakatsuji, H., Gaussian 16. In *Revision A*, Gaussian, Inc., Wallingford CT: 2016; Vol. 3.
3. Zhao, Y.; Truhlar, D. G., The M06 suite of density functionals for main group thermochemistry, thermochemical kinetics, noncovalent interactions, excited states, and transition elements: two new functionals and systematic testing of four M06-class functionals and 12 other functionals. *Theor. Chem. Acc.* **2008,** *120* (1), 215-241.
4. Binkley, J. S.; Pople, J. A.; Hehre, W. J., Self-consistent molecular orbital methods. 21. Small split-valence basis sets for first-row elements. *J. Am. Chem. Soc.* **1980,** *102* (3), 939-947.
5. Lee, C. T.; Yang, W. T.; Parr, R. G., Development of the Colle-Salvetti Correlation-Energy Formula into a Functional of the Electron-Density. *Phys. Rev. B* **1988,** *37* (2), 785-789.
6. Becke, A. D., Density‐functional thermochemistry. III. The role of exact exchange. *J. Chem. Phys.* **1993,** *98* (7), 5648-5652.
7. Hehre, W. J.; Ditchfield, R.; Pople, J. A., Self—Consistent Molecular Orbital Methods. XII. Further Extensions of Gaussian—Type Basis Sets for Use in Molecular Orbital Studies of Organic Molecules. *J. Chem. Phys.* **1972,** *56* (5), 2257-2261.
8. Petersson, G. A.; Al‐Laham, M. A., A complete basis set model chemistry. II. Open‐shell systems and the total energies of the first‐row atoms. *J. Chem. Phys.* **1991,** *94* (9), 6081-6090.
