# Supplementary material for: Synthesis and assembly of extended quintulene
Source: Nat Commun. 2020 Aug 7;11:3976. doi: 10.1038/s41467-020-17691-7 (PMC7414228; doi:10.1038/s41467-020-17691-7)
Supplement: Supplementary file 3 — Description of Additional Supplementary Files [file 41467_2020_17691_MOESM3_ESM.pdf]

Description of Additional Supplementary Files

Supplementary Data 1. Cartesian coordinates of monomeric **1**.

Supplementary Data 2. Cartesian coordinates of dimeric (**1**)$_2$.

Supplementary Data 3. Cartesian coordinates of counterpart of **1** without the cavity.
